# Specifically targeting ERK1 or ERK2 kills Melanoma cells

**DOI:** 10.1186/1479-5876-10-15

**Published:** 2012-01-25

**Authors:** Jianzhong Qin, Hong Xin, Brian J Nickoloff

**Affiliations:** 1 Department of Microbiology and Immunology, University of Illinois at Chicago Medical Center, Chicago, IL 60612, USA; 2 Department of Obstetrics and Gynecology, Northwestern University School of Medicine Feinberg School of Medicine, Chicago, IL 60611, USA; 3 Division of Dermatology, Michigan State University, College of Human Medicine, Grand Rapids, MI 49503, USA; 4 Center for Cancer Genomics and Computational Biology, Laboratory of Cutaneous Oncology, Van Andel Research Institute, 333 Bostwick Ave NE, Room 5008, Grand Rapids, MI 49503-2518, USA

**Keywords:** ERK, Melanoma, Drug resistance, BRAF, PLX4032

## Abstract

**Background:**

Overcoming the notorious apoptotic resistance of melanoma cells remains a therapeutic challenge given dismal survival of patients with metastatic melanoma. However, recent clinical trials using a BRAF inhibitor revealed encouraging results for patients with advanced BRAF mutant bearing melanoma, but drug resistance accompanied by recovery of phospho-ERK (pERK) activity present challenges for this approach. While ERK1 and ERK2 are similar in amino acid composition and are frequently not distinguished in clinical reports, the possibility they regulate distinct biological functions in melanoma is largely unexplored.

**Methods:**

Rather than indirectly inhibiting pERK by targeting upstream kinases such as BRAF or MEK, we directly (and near completely) reduced ERK1 and ERK2 using short hairpin RNAs (shRNAs) to achieve sustained inhibition of pERK1 and/or pERK2.

**Results and discussion:**

Using A375 melanoma cells containing activating BRAF^V600E ^mutation, silencing ERK1 or ERK2 revealed some differences in their biological roles, but also shared roles by reduced cell proliferation, colony formation in soft agar and induced apoptosis. By contrast, chemical mediated inhibition of mutant BRAF (PLX4032) or MEK (PD0325901) triggered less killing of melanoma cells, although they did inhibit proliferation. Death of melanoma cells by silencing ERK1 and/or ERK2 was caspase dependent and accompanied by increased levels of Bak, Bad and Bim, with reduction in p-Bad and detection of activated Bax levels and loss of mitochondrial membrane permeability. Rare treatment resistant clones accompanied silencing of either ERK1 and/or ERK2. Unexpectedly, directly targeting ERK levels also led to reduction in upstream levels of BRAF, CRAF and pMEK, thereby reinforcing the importance of silencing ERK as regards killing and bypassing drug resistance.

**Conclusions:**

Selectively knocking down ERK1 and/or ERK2 killed A375 melanoma cells and also increased the ability of PLX4032 to kill A375 cells. Thus, a new therapeutic window is open for future clinical trials in which agents targeting ERK1 and ERK2 should be considered in patients with melanoma.

## Background

The incidence of melanoma is on the rise [1], as is the number of individuals dying from metastatic melanoma [2]. There are numerous genetically defined activating mutations in melanoma cells leading to enhanced activity of the RAF/MEK/ERK signaling cascade [3-7]. Numerous recent reports focusing on BRAF-targeted therapy designed to interrupt the RAF/MEK/ERK mitogen activated protein kinase (MAPK) pathway in melanoma patients have not made any distinctions between ERK1 and ERK2 [8-15]. To our knowledge no group has attempted to distinguish or target the different isoforms of ERK (e.g. ERK1 or ERK2) specifically in melanoma cells (reviewed in [16]).

Over 20 years ago, it was discovered that a prominent response to addition of extracellular mitogen to fibroblasts triggered a series of intracellular biochemical events including several kinases such as MEK and p44^MAPK^/ERK1 [17-20] and p42^MAPK^/ERK2 [20]. While ERK1 and ERK2 share 84% amino acid sequence homology, knocking out ERK1 vs. ERK2 in mice produces different phenotypes supporting distinct functions for these isoforms [21,22]. Many components of RAF/MEK/ERK signaling cascades are mutated or aberrantly expressed in human cancer cells responsible for transformation accompanied by altered proliferation, survival and resistance to treatment [23]. As clinicians have refocused their therapeutic strategies including targeting mutated BRAF, and downstream molecules such as MEK, the potential efficacy of targeting ERK1 and/or ERK2 has not been tested [24].

To fill the experimental and therapeutic void regarding the roles for ERK1 and/or ERK2 in human melanoma, a cell line containing mutated BRAF (e.g. A375 cells) was studied in detail using shRNAs selective for each isoform. After confirming effective and selective silencing of ERK1 and ERK2, a series of experiments was conducted to evaluate these kinases in melanoma. While functional differences between ERK1 and ERK2 are controversial depending on the cell type examined [25], we observed both similar as well as distinct effects such as differentially involving specific pro-apoptotic proteins (i.e. Noxa) in A375 cells upon silencing of ERK1 and ERK2. Given that activation of the ERK pathway is important in melanoma progression [26], these findings lay the groundwork for new approaches in metastatic melanoma using a molecularly-based targeted approach [27].

Such novel approaches are urgently needed as it is clear that melanoma cells possess multiple mechanisms to bypass, or overcome drug resistance to agents with clinical success such as PLX4032 (Vemurafenib), a drug targeting mutant BRAF [9,28]. An interesting and relevant common intersection point for the various roads to PLX4032 resistance is ERK signaling (ibid). Thus, we decided to expand our studies to not only include silencing of ERK1 and/or ERK2, but to compare and contrast the biological responses and bypass mechanisms triggered by exposing A375 melanoma cells to PLX4032, as well as a MEK inhibitor (PD0325901). The results clearly demonstrate that not only is a combination of ERK1 and ERK2 superior in triggering a caspase-dependent mode of killing A375 melanoma cells compared to PLX4032 or PD0325901, but drug resistant clones infrequently appear by directly targeting ERK. The ability of using ERK shRNAs to not only kill melanoma cells, but to block emergence of treatment resistant clones likely involves not only reductions in levels of phospho-ERKs, but also in upstream reductions in BRAF, CRAF and phospho-MEK thereby interrupting a feedback loop critical to melanoma survival. ERK shRNAs were also shown to increase the sensitivity of melanoma cells to killing by PLX4032 paving the way for combination therapeutic approaches in melanoma. These results demonstrate that targeting ERK in melanoma can overcome the apoptotic resistance of this highly aggressive and difficult to cure tumor.

## Methods

### Cell culture and chemicals

The human melanoma cell line A375 was purchased from American Type Culture Collection (Manassas, VA, USA) and maintained in DMEM (Lonza, Walkersville, MD, USA) plus 10% FCS (Gemini Bio-Products, Woodland, CA) in a humidified incubator (37°C, 5% CO_2_). Annexin-FITC was purchased from Biovision Research Products (Mountain View, CA, USA), and tetra methyl rhodamine ethyl ester (TMRE) was purchased from Invitrogen Molecular Probes (Eugene, OR, USA). Pan-caspase inhibitor ZVAD was purchased from BD Biosciences (San Jose, CA, USA). Propidium iodide (PI) was purchased from Sigma Chemical Co (St Louis, MO, USA). PD0325901 and PLX4032 were purchased from Biovision Research Products (Mountain View, CA, USA) and Selleck Chemicals (Houston, TX, USA), respectively. Abs against ERK1, ERK2, pERK1, pERK2, MEK, pMEK, p-Bad, Bak, Bim, PUMA were purchased from Cell Signaling Technology (Beverly, MA, USA); whereas Bcl-XL, Mcl-1, Bad, PARP, caspase 3, Raf-1, Raf-B and GAPDH were purchased from Santa Cruz (Santa Cruz, CA). Ab against Bcl-2 was from DAKO (Glostrup, Denmark), ab against actin from Chemicon Int. (Billerica, MA, USA); Ab against Bax from Calbiochem (San Diego, CA, USA), and against XIAP and activated Bax from BD Transduction Lab (Franklin Lakes, NJ, USA). Primary Abs incubated overnight at 4°C, and secondary Abs were incubated at room temperature for 1 hr.

### Production of lentiviral supernatants

Mission TCR shRNAs targeting human ERK1 (NM_002746) and ERK2 (NM_138957) were purchased from Sigma Chemical Co. pLKO.1 Scramble control shRNA plasmid, psPAX2 packaging plasmid and pMD2.G envelope plasmid were provided by Addgene. To make lentiviral particles, HEK-293 T cells were plated into 10 cm plates, 2 × 10^6 ^cell/plate, with 8 ml of DMEM plus 10% FBS and no antibiotics. On the next day, for each plate 3 ug of pLKO.1 shRNA plasmid together with 2.25 ug of psPAX2 and 0.75 ug of pMD2.G plasmid were transfected with FuGen 6 reagents (Roche, New Jersey) according to the manufacture's instruction. The transfection reagent was removed by replacing the medium with fresh DMEM containing FBS and penicillin/streptomycin on the following day. The cells were incubated at 37°C, 5% CO_2 _for 24 hr for another 2 days. Supernatants from 24 hr and 48 hr incubations were harvested and combined followed by centrifugation to remove cell debris and stored at -80°C.

### Gene transduction with lentivirus based shRNA

A375 melanoma cells were plated onto 6 well plates at 3 × 10^5 ^cells/well and incubated at 37°C, 5% CO_2 _overnight. Cells were washed 1x with PBS and 1 ml of lentiviral supernatants containing shRNA for either ERK1 or ERK2 or scramble control was added in each well. For ERK1 and ERK2 double knockdown, both supernatants (1 ml of each) were added into one well. All viral supernatants were added with hexadimethrine bromide (Sigma Chemical Co.), final concentration 8 μg/ml) before use. After 4-6 hr incubation, supernatants were changed with fresh medium and cells were incubated for another 1 or 2 days before being split for experiments.

### Quantitation of cell viability

Cell death was measured by flow cytometry after staining cells with Annexin-V-FITC and I mg/ml of PI. To investigate DNA degradation, one of the hallmarks of apoptosis, cells were fixed with 70% ethanol and PI stained in the presence of RNAse (10 μg/ml). The relative percentage of cells with hypo-diploid DNA content (sub-G_0_) was determined by FACS analysis and use of Excel software.

### Colony formation assays

For anchorage independent colony formation assay, A375 cells were transduced with shRNAs for 2 days and then suspended in DMEM with 0.5% agarose solution, and plated onto solidified 1% agarose in 6 well plate at a density of 2500 cells per well in triplicate. A375 cells were maintained in culture by feeding with 0.5 ml fresh DMEM plus 10% FBS medium twice a week, for a total 3 weeks. Two independent assays were carried out and the number of colonies were counted after staining with 0.1% crystal violet solution at the end of each experiment. For anchorage dependent colony assay, A375 cells infected with shRNAs for 2 days were seeded into 6 well plates at a density of 2000 cells per well in triplicate. The cells were cultured with complete DMEM for another 9 days and colony number counted after being stained with 0.1% crystal violet solution.

### Mitochondrial membrane potential assay

Assessment of mitochondrial membrane potential was determined by addition of 100 nM of TMRE dye that accumulates in mitochondria of living cells [29]. Reduction in TMRE retention is indicative of loss of mitochondrial membrane potential.

### Detection of intracellular levels of activated BH3-multidomain proapoptotic bax protein

A375 cells were fixed with 2.5% paraformaldehyde (10 min, room temperature), washed and incubated with a primary ab detecting the activated configuration of Bax (BD Pharmingen Inc, San Diego, CA, USA) in FACS buffer with 0.3% saponin as previously described [30]. The percentage of melanoma cells with activated Bax was measured by fluorescence intensity greater than control ab levels.

### Immunoblotting

Western blot analysis was performed as previously described [31]. Briefly, cells were harvested by scraping and lysed with M-Per mammalian protein extraction reagent (Thermo Scientific, Rockville, IL, USA) supplemented with protease inhibitor cocktail (Roche Diagnostics GmbH, Germany) and phosphatase inhibitor cocktail set II, (Calbiochem, Los Angeles, CA, USA) followed by shaking and centrifugation at 4°C. Supernatants were collected as whole cell extracts and protein concentrations were measured using Bradford reagents (Bio-Rad laboratories, Hercules, CA, USA). 30 ug of proteins were resolved by SDS- PAGE and transferred to PVDF membrane followed by 1 hr blocking with buffer supplied by LI-COR Biosciences (Lincoln, NE, USA). Blots were probed with primary Abs overnight at 4°C, washed and incubated with corresponding fluorescence-labeled secondary Ab for 1 hr at room temperature in dark. Protein levels were visualized with LI-COR Infrared Imaging System.

### Reverse transcriptase-real time PCR

Total RNA was extracted from A375 cells using Trizol (Invitrogen Life Technologies, Inc.). One microgram of RNA was reverse transcribed to cDNA using TaqMan reverse transcription reagents (Applied Biosystems, Foster City, CA). The following specific primer pairs were used: BRAF: 5'-CTC GAG TGA TTG GGA GAT TCC TGC-3', (forward), 5'-CTG CTG AGG TGT AGG TGC TGT CAC-3' (reverse); 18sRNA: 5'-GGC GCC CCC TCG ATG CTC TTA G-3', (forward), 5'-GCT CGG GCC TGC TTT GAA CAC TCT-3', (reverse). PCR reaction was performed by adding 25 μl of 2X SYBR Green Supermix (Bio-Rad Laboratories, Hercules, CA, USA), 19 μl DEPC treated H_2_O, 2 μl of each primer, 2 μl of diluted cDNA template. DNA amplification was completed in ABI prism model 7300 thermal cycler. All reactions were run in triplicate and two independent assays were performed. The comparative expression level was determined by applying the calculation of 2^(Δ Ct B-raf-Δ*Ct *18s).^

### Statistical analysis

All statistical analyses were performed using the unpaired, two sided Student's *t*-test, and results considered significant when *P *values were less than 0.05.

## Results

### ERK1 and ERK2 shRNA selectively knockdown ERK1 and ERK2 proteins, respectively in A375 melanoma cells, accompanied by killing of cells involving apoptosis

**Figure 1:**
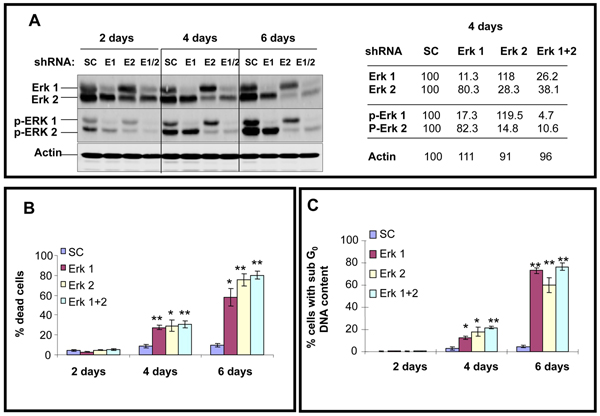
**ERK1 and/or ERK2 shRNA treatment of A375 cells selectively reduces total and phosphorylated levels of respective proteins at days 2, 4 and 6; accompanied by killing of melanoma cells**. **A**. Western blot showing total and phosphorylated protein levels at indicated time intervals following exposure to shRNAs as indicated. SC: scrambled control; E1: ERK1; E2: ERK2; E1/2: ERK1 and ERK2. Actin levels confirm equal loading (left side panel). Data presented is representative of three independent experiments. Right side panel shows the relative density measurement of day 4 treatment levels for indicated proteins. **B**. Relative killing percentages on days 2, 4, and 6 following exposures to indicated shRNAs. Percentage of dead cells as described in *Methods *section using Annexin-PI and FACS analysis. Histograms represent the means +/- SEM from four independent experiments. Statistical significance portrayed as follows: *p < 0.002; **p < 0.0001. **C**. Relative killing percentages on days 2, 4, and 6 following exposures to indicated shRNAs. Percentage of cells undergoing apoptosis (cells with sub-Go DNA content) as described in *Methods *section Histograms represent the means +/- SEM from three independent experiments. Statistical significance portrayed as follows: *p < 0.03; **p < 0.001.

In A375 cells, constitutive levels for ERK2 are slightly greater than ERK1, and the pERK2 is also slightly more abundant compared to pERK1 (Figure 1A). As there are currently no chemical inhibitors to selectively block ERK1 versus ERK2 activity [32], lentiviral preparations containing specific shRNAs were used targeting ERK1 or ERK2. Addition of ERK1 shRNA significantly reduced protein levels of ERK1 and pERK1 compared to scrambled control shRNA treatment, as observed beginning on day 2, with greater reduction on day 4 and near complete absence on day 6. Interestingly, reduction in ERK1 levels was accompanied by minimal changes in the protein level of ERK2 and pERK2 (Figure 1A, left side panel). Similarly, addition of ERK2 shRNA significantly reduced protein levels of ERK2 and pERK2 compared to scrambled control shRNA treatment with similar kinetics as observed for ERK1 shRNA, with minimal changes in Erk1 and pErk1 protein levels by the ERK2 shRNA. Quantitative analysis of a representative blot on day 4 is presented in Figure 1A-right side panel. Thus, despite both ERK1 and ERK2 are MEK substrates; significant reductions in one isoform did not trigger increased phosphorylation of the other isoform in A375 cells.

Treatment with shRNAs silencing ERK1 and/or ERK2 did not trigger detectable killing of A375 cells 2 days after infection (although G_1 _growth arrest was noted, data not shown), but progressively increased killing was observed 4 days (*p *< 0.0001 for ERK1 and ERK1 plus ERK2 shRNAs; and *p *< 0.002 for ERK2 shRNA) and 6 days (*p *< 0.002 for ERK1 shRNA and *p *< 0.001 for ERK2 and ERK1 plus ERK2 shRNAs) after infection (Figure 1B). Note by combining ERK1 plus ERK2 shRNA infections, no significant additive or synergistic increased killing was observed. To determine the mode of cell death cells with sub G_0 _DNA content were also analyzed and found to be increased (*p *< 0.03 for ERK1 or ERK2 shRNAs at 4 days), and *p *< 0.001 for ERK1 plus ERK2 shRNA at 6 days pointing to a role for apoptosis in the response to the ERK shRNAs (Figure 1C).

### ERK1 and ERK2 shRNA triggers reduction in soft agar colony formation and lack of drug resistance

**Figure 2:**
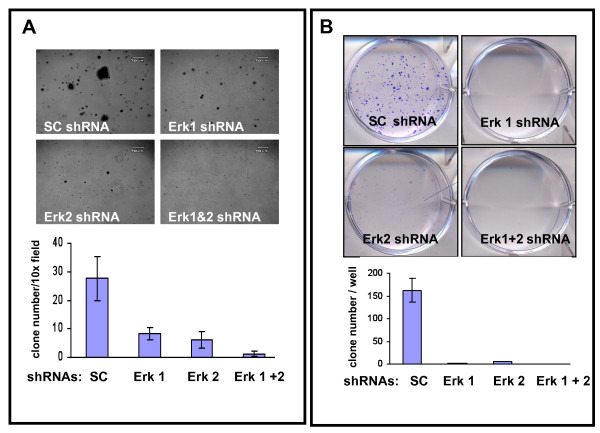
**ERK1 and/or ERK2 shRNA treatment of A375 cells reduces clonogenic expansion and growth of colonies**. **A**. Colony formation in soft agar after 21 days using A375 cells exposed to shRNAs (upper panel-phase contrast microscopic appearance after crystal violet staining). Quantification of clone number per 10x microscopic field for cells exposed to shRNAs for duplicate experiments performed with triplicate wells. (lower panel; all values using ERK1 and/or ERK2 shRNA compared to SC shRNA, *p *< 0.001). **B**. Appearance of A375 cells after exposure (9 days) to indicated shRNAs revealing crystal violet stained clones readily visible in the SC shRNA treated cultures, but rare to absent in the other wells (upper panel). Quantitation of clone number per well for A375 cells exposed for 9 days to indicated shRNAs for duplicate experiments performed with triplicate wells (lower panel; all values using ERK1 and/or ERK2 shRNA compared to SC shRNA, *p *< 0.001).

Next, the biological effect of a sustained reduction in pERK1 and/or pERK2 on anchorage independent colony formation in soft agar (Figure 2A) was determined (21 days). There was significant reduction in melanoma cell colony forming potential followed treatment with ERK1 shRNA or ERK2 shRNA; with virtually no colonies forming following combined ERK1 plus ERK2 shRNAs (all values *p *< 0.001) as assessed by counting the number of clones per 10X magnification light microscopy (Figure 2A lower panel).

To assess potential of anchorage dependent colony formation, clones of A375 melanoma cells developing resistance to ERK1 and/or ERK2 shRNA treatment were determined by visual inspection and manual counting of the numbers of clones developing after 9 days (Figure 2B). While numerous and easily visible clones were apparent when cells were treated with SC shRNA, treatment with the ERK shRNAs did not generate any resistant clones when used in combination, and only rare clones could be identified with single treatment, being greater for ERK2 shRNA than ERK1 shRNA as assessed by counting the number of clones per well (Figure 2B lower panel; all reductions *p *< 0.001).

### Knockdown of ERK1 and/or ERK2 triggers killing of A375 melanoma cells involving a caspase dependent cascade

**Figure 3:**
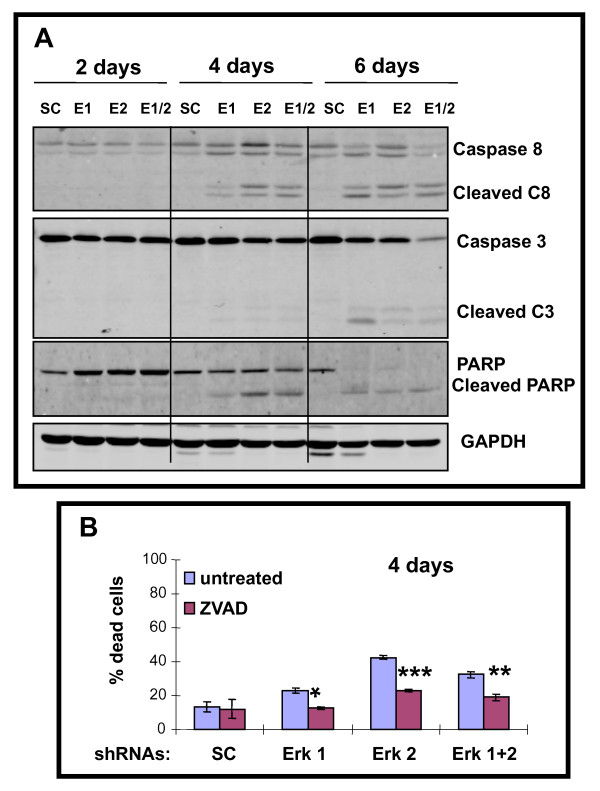
**ERK1 and/or ERK2 shRNA triggers caspase cascade including PARP cleavage, and caspase inhibitor partial block of killing of A375 cells**. **A**. Western blot analysis of caspase 3, caspase 8 and PARP including both intact (inactive) and cleaved (active) forms in A375 cells on days 2, 4 and 6 following exposure to shRNAs. GAPDH confirms equivalent loading. **B)**. Addition of pan-caspase inhibitor, ZVAD (20 mM; last 2 days) reduces killing of A375 cells triggered by ERK1 and/or ERK2 shRNA. Histograms represent the means +/- SEM from 3 independent experiments. Statistical significance portrayed as follows: *p < 0.01, **p < 0.02, ***p < 0.002.

Western blot analysis revealed silencing either ERK1 and/or ERK2 triggered cleavage (activation) of caspase 8 after 4 days and 6 days, with cleaved caspase 3 detected after 6 days of treatment (Figure 3A). PARP was cleaved more prominently by ERK2 shRNA compared to ERK1 shRNA (similar to caspase 8) on day 4, and by day 6 ERK1 and/or ERK2 shRNAs triggered near complete cleavage of PARP. The role of caspases in killing of A375 cells was studied using the pan-caspase inhibitor, ZVAD (Figure 3B); which revealed significant blocking of day 4 cell death by ERK1 shRNA (*p *< 0.01; *p *< 0.002 for ERK2 shRNA and *p *< 0.02 for ERK1 plus ERK2 shRNAs).

### Killing of A375 cells involves alterations in mitochondrial function

**Figure 4:**
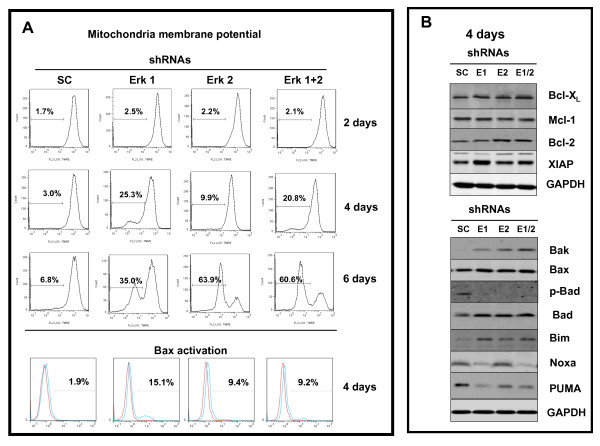
**Decreased mitochondrial outer membrane potential by silencing ERK1 and/or ERK2; accompanied by increased levels of activated Bax**. **A**. Treatment with ERK1 and/or ERK2 shRNAs at indicated time points increase outer mitochondrial membrane permeability as assessed by TMRE labeling as described in Methods (upper panel). Increased presence of activated Bax detected in A375 cells treated for 4 days with ERK1 and/or ERK2 shRNA (lower panel). **B**. Western blot analysis of selected proteins mediating pro-survival (upper panel) versus pro-apoptosis (lower panel) in A375 cells exposed for 4 days with either shRNAs. GAPDH levels confirm equivalent protein loading.

To assess the integrity of the outer mitochondrial membrane potential before and after 2, 4 and 6 days of treatment, A375 cells were labeled with a TMRE dye (Figure 4A), and reduction in the intensity of staining indicating altered membrane potential was progressively increased over the time course for ERK1, ERK2 and ERK1 plus ERK2 shRNAs in A375 cells. Further analysis using abs detecting the activated forms for Bax in permeabilized cells with FACS (Figure 4A-lowest panel set), revealed all shRNA treatments triggered increased levels of activated Bax within the melanoma cells.

### Profiling protein levels regulating survival and death in A375 cells

Since cell death is regulated by relative levels of various pro-apoptotic and anti-apoptotic proteins (Figure 4B), cell extracts were prepared before and 4 days after various treatments and western blot analysis included a panel of anti-apoptotic proteins (Figure 4B upper panel), and pro-apoptotic proteins (Figure 4B lower panel). Constitutive levels for proteins associated with cell survival were detected for Bcl-X_L_, Mcl-1, Bcl-2, and XIAP (Figure 4B upper panel). Treatment with ERK1 and/or ERK2 shRNA did not lower these levels. There was slight increase in XIAP by knocking down ERK1 but not ERK2, which did not seem to protect cells from the killing by targeting ERK2. As regards the pro-apoptotic protein levels (Figure 4B lower panels), knockdown of ERK1 and/or ERK2 increased levels of Bak, Bax, Bad, Bim with variable changes in Noxa and PUMA levels, accompanied by decreased p-Bad levels in the A375 cells. Both Noxa and PUMA appeared to be dependent on ERK1, consistent with recent report [33]. Overall, targeting ERKs appeared to more significantly influence pro-apoptotic protein levels over pro-survival protein levels.

### PLX4032 and PD0325901 rapidly reduce pERK1 and pERK2, but pERK1/2 levels are restored following PLX4032 treatment, with killing beginning on day 2 but without significant increases after 4 and 6 days of treatment

**Figure 5:**
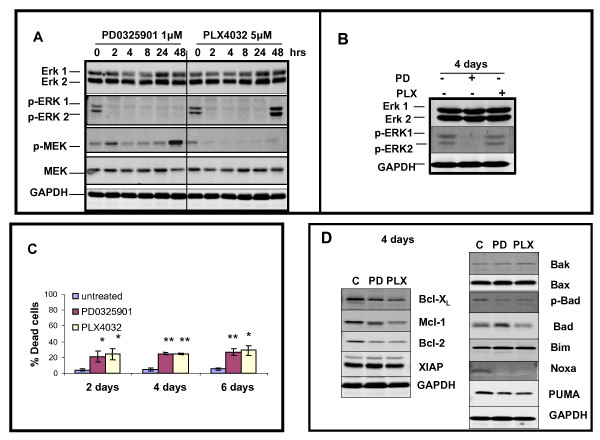
**PD0325901 or PLX4032 treatment of A375 melanoma cells triggers rapid reduction in pERK1 and pERK2 levels but these levels are restored after 4 days with PLX4032; accompanied by increased killing, and alterations in protein levels regulating survival and apoptosis**. **A**. Kinetic changes of selected protein levels by immunoblot analysis altered by treatment with PD0325901 (1 μM) or PLX4032 (5 μM) for 2, 4, 8, 24, 48 hr of drug exposure (left side panel). **B**. Similar analysis of protein levels treated with PD0325901 or PLX4032 after 4 days of drug exposure (right side panel). GAPDH levels confirm equal protein loading. **C**. Relative killing percentages of A375 cells on days 2, 4, and 6 following exposure to PD0325901 (1 μM) or PLX4032 (5 μM). Percentage of dead cells as described in *Methods *section. Killing at 2 days by either drug (*p *< 0.01), after 4 days (*p *< 0.001) and after 6 days (PD0325901, *p *< 0.001; PLX4032, *p *< 0.01) based on 3 independent experiments. Statistical significance portrayed as follows: *p < 0.01, **p < 0.001. **D**. Western blot analysis of selected proteins mediating pro-survival (left side panel) versus pro-apoptosis (right side panel) in A375 cells exposed for 4 days to PD0325901 (1 μM) or PLX4032 (5 μM). GAPDH indicates equivalent protein loading.

Treatment with the MEK inhibitor, PD0325901 reduced ERK1 levels at 4 hr and 8 hr, but eliminated pERK1 and pERK2 levels between 2 hr and 48 hr (Figure 5A), with sustained reduction after 4 days (Figure 5B). Treatment with mutant BRAF specific inhibitor, PLX4032 slightly influenced ERK1 and ERK2 levels, but eliminated pERK1 and pERK2 levels up to 24 hr; but this was not sustained as both phosphorylated isoforms were detectable after 48 hr and 4 days (Figure 5A, B, respectively). Induction of A375 cell killing was rapid (Figure 5C) as detected at the 2 day assay point, (approximately 20% of the cell population; *p *< 0.01 for both drugs), with slight increases on day 4 (*p *< 0.001 for both drugs) or day 6 (*p *< 0.001 for PD0325901 compound and *p *< 0.01 for PLX4032).

PD0325901 treatment triggered slight reductions in Bcl-X_L_, Mcl-1, Bcl-2 after 4 days of treatment, but not XIAP; whereas PLX4032 triggered greater reductions all of these pro-survival protein levels, but not XIAP (Figure 5D). Pro-apoptotic protein levels were either relatively unchanged or diminished by treatment with these two drugs, which may reflect the overall relatively modest levels of killing achieved during the six-day period of treatment with PD032509 or PLX4032.

### Silencing ERK1 and/or ERK2 reduces upstream kinase levels including BRAF, CRAF, and pMEK

**Figure 6:**
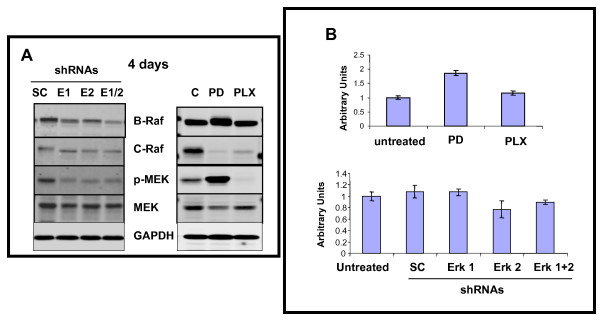
**Silencing ERK1 and/or ERK2 reduces protein levels of upstream kinases after 4 days including BRAF, CRAF, and phospho MEK; while PD0325901 or PLX4032 exposure variably influences these kinases, with less to no effects of any treatments on BRAF mRNA levels**. A. Western blot of upstream kinases in A375 cells 4 days after treatment with shRNAs (left side panel), or after exposure to PD0325901 (1 μM) or PLX4032 (5 μM) on right side panel. GAPDH confirms equivalent protein loading. B. BRAF mRNA levels after 4 days of exposure to PD0325901 orPLX4032 (upper panel); or after treatment with shRNAs (lower panel). Histograms represent the means +/- SD based on three independent experiments.

To explore the molecular basis for lack of emergence of treatment resistance clones when using ERK1 and/or ERK2 shRNAs, relative levels of upstream kinases were determined 4 days after treatment (Figure 6A-left side panel). Compared to levels observed using SC shRNA, the levels of BRAF, CRAF, phospho-MEK, but not total MEK were significantly reduced by either ERK1 shRNA or ERK2 shRNA, and for BRAF became even lower with ERK1 and ERK2 shRNA combined. Given the reports of treatment resistance of melanoma cells to PLX4032, we compared and contrasted the same upstream kinase levels after 4 days of treatment with either PLX4032 or PD0325901 (Figure 6A-right side panel). Compared to untreated A375 cells, continuous exposure to PD0325901 lead to increased levels of BRAF, which was also modified so as to reduce its electrophoretic motility, accompanied by near complete loss of CRAF and increased phospho-MEK and slightly reduced total MEK levels. By contrast, PLX4032 treatment did not reduce BRAF levels, although both CRAF and phospho-MEK levels were dramatically reduced. Since BRAF levels appeared to discriminate between the shRNA treatment that suppressed resistant clone formation and PLX4032 that promoted resistant clone formation, relative mRNA levels were determined using RT-PCR (Figure 6B). Compared to untreated cells, PD0325901 increased BRAF mRNA levels, but none to relatively minor changes in BRAF mRNA levels were observed for all other treatments (PLX4032, ERK1 and/or ERK2 shRNAs), indicating changes involving non-transcriptional regulatory elements.

### Combining ERK shRNAs with PLX4032 enhances killing of melanoma cells

**Figure 7:**
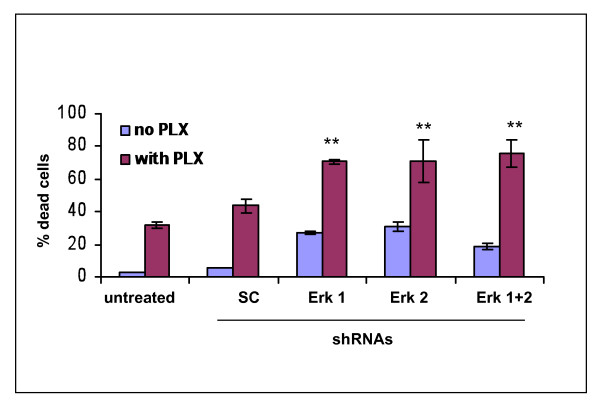
**Silencing ERK1 and/or ERK2 increase sensitivity of A375 cells to killing by PLX4032**. **A**. Relative killing percentages of A375 cells by PLX4032 (5 mM; 4 days) alone or when combined with indicated shRNAs. A375 cells were infected with shRNAs for a total of 4 days and PLX4032 added for the last 2 days. Note compared to either PLX4032 alone or the shRNAs alone, when combined there is increased killing (*p *< 0.001). Histograms represent the means +/- SEM based on 3 independent experiments. Statistical significance portrayed as follows: **p < 0.001.

As noted in Figure 5A, treatment of A375 cells with PLX4032 led to a rebound in pERK levels after 48 hrs, and to determine if this rebound level of pERK contributed to melanoma treatment resistance associated with PLX4032 treatment, we first knocked down pERK levels with the ERK shRNAs (which generate sustained and selectively knockdown of each pERK isoform (Figure 1A), and then added PLX4032. Compared to the killing of A375 cells accomplished by either use of ERK shRNAs or PLX4032 alone (Figure 7), greater killing was triggered by PLX4032 in the co-presence of ERK1 shRNA, ERK2 shRNA or ERK1 plus ERK2 shRNA (*p *< 0.001 for all combinations compared to single treatment values).

## Discussion and conclusions

Despite an impressive array of exciting recent results highlighting the importance of the RAF/MEK/ERK pathway in melanoma patients, surprisingly these reports did not make any distinctions between ERK1 and ERK2. By contrast, we focused on dissecting important similarities and differences in the biology and therapeutic targeting efficacy between ERK1 and ERK2 in A375 melanoma cells. Taken together, the detailed characterization of the cellular and molecular events in A375 melanoma cells following silencing of ERK1 and/or ERK2 revealed many insights of potential therapeutic significance. First, it is clear that ERK1 and ERK2 control similar, but not identical, signaling events in melanoma cells. For example, reducing ERK2, but not ERK1 increased levels of Noxa; yet combining ERK1 plus ERK2 shRNAs reduced Noxa below constitutive levels; whereas reducing ERK1 but not ERK2 increased XIAP levels (Figure 4B). Also, while both isoforms compete for MEK, reduction in one isoform did not lead to increased phosphorylation of the other isoform in A375 cells (Figure 1A). Second, targeting ERK1 and/or ERK2 trigger greater cell death in A375 cells compared to chemical inhibitors of mutant BRAF (PLX4032) or MEK (PD0325901) that possessed similar rapid reductions in phospho-ERK levels (Figures 1A, B, and 5A, B).

Besides the aforementioned attributes of targeting ERK1 and/or ERK2, there are several other advantages to this strategy. First, since drug resistance to PLX4032 is linked to recovery of pERK activity [34], it was therapeutically beneficial that sustained and near complete reduction in both pERK1 and pERK2 can be accomplished using the shRNAs to obviate this problem. Second, along the same line of inquiry, unlike PLX4032 which is preferentially active in tumor cells bearing the V600E mutation of BRAF, reducing levels of pERK1 and/or 2 does not depend on the mutation status of the melanoma cell. Indeed, it has been observed that within melanoma lesions, there can be both clones containing both BRAF mutant alleles as well as wild-type BRAF [35]. Third, by silencing ERK1 and/or ERK2, the feedback loop between ERK and RAF and MEK is interrupted as clearly observed by comparing treatment of cells with MEK inhibitor which led to increased phospho-MEK as reported by others [36], in contrast to the decrease in phospho-MEK after our shRNA approach (see below for more discussion).

Additional evidence for feedback loops, and significant molecular complexities became apparent by probing for changes in BRAF and CRAF with these various treatments. It should be noted that phosphorylation events amongst the components of this pathway can be either activating or inhibitory [37]. A simple negative feedback loop has been suggested whereby activated ERK would phosphorylate and thereby inhibit further MEK activity [38]. While blocking ERK with the chemical inhibitors or the ERK shRNAs would therefore be expected to enhance phospho-MEK levels, this was only observed using PD0325901 (Figure 6A). Thus, feedback loops are more complicated as regards ERK and MEK. In another scenario, downstream from ERK are phosphatases (e.g. DUSP) that serve as negative feedback components, and decreased ERK levels could lead to reduced phosphatases thereby facilitating accumulation of phospho-MEK [39].

Perhaps even greater complexity was uncovered between ERK and more upstream kinases such as BRAF and CRAF. In this situation, markedly diminished levels of BRAF and CRAF accompanied using shRNAs, reducing ERK1 and or ERK2 levels. ERK is known to be able to hyperphosphorylate members of the RAF serine/threonine kinase family, leading to decreased signaling, and it is possible the altered phosphorylation triggered by decreased ERK levels influenced the ability of the antibody to recognize BRAF or CRAF due to conformational changes [38]. Support for this posttranscriptional modification was provided by the RT-PCR results in which the shRNAs did not influence relative mRNA levels for BRAF (Figure 6B). A feedback loop could also be observed using the MEK inhibitor as regards increased BRAF levels and decreased electrophoretic motility possibly due to hyperphosphorylation [40,41]. While undoubtedly complex, elements of the feedback loops (both positive and negative) will require exploration beyond the scope of this current work, but are likely contributors to the lack of emergence of treatment resistant clones using the ERK shRNAs.

Despite the remaining challenges required to more fully understand the biology of silencing ERK1 and or ERK2, it is clear that killing of melanoma cells by silencing ERK1 and/or ERK2 is caspase dependent (Figure 3B), provoking altered mitochondrial function (Figure 4A), and highlights a key role for the ERK signaling pathway in tumor cell survival [42]. The higher level of cytotoxicity exhibited by the ERK shRNAs and activation of caspase cascades also are likely to contribute to the paucity of treatment resistant clones (Figure 2B) with our novel therapeutic approach. Furthermore the ability of PLX4032 to kill A375 melanoma cells was greatly increased when combined with ERK1 and/or ERK2 shRNAs (Figure 7), suggesting the possibility of such combination therapies worthy of a additional study.

Based on the current in-vitro findings, it may be worth moving forward in the clinic with several drug combinations to prevent drug resistance to PLX4032, which would include drugs targeting ERK, or with drugs targeting MEK. While these findings were consistently observed in the A375 cell line, future studies are indicated to explore other V600E BRAF mutant bearing cell lines to compare and contrast with the results for A375 cells. In conclusion, targeting the BRAF-MEK-ERK pathway for melanoma patients is rapidly accelerating in the clinic [43,44], and further studies using agents that silence ERK1 and/or ERK2 should be seriously considered for future lines of inquiry to overcome the notorious apoptotic resistance and treatment bypass repertoire of melanoma cells either alone or in combination with PLX4032 [45].

## Abbreviations

Ab: Antibody; ERK: Extracellular-signal-regulated kinase; FCS: Fetal calf serum; GAPDH: Glyceraldehyde 3 phosphate dehydrogenase; sh: Short hairpin; MAPK: Mitogen-activated protein kinase; MEK: MAPK kinase/ERK kinase; PI: Propidium iodide; cDNA: complimentary deoxyribonucleic acid; Ab: antibody; DUSP: dual specificity phosphatases; RT-PCR: Reverse transcriptase-polymerase chain reaction; TME: Tetramethylrhodamineethylester.

## Competing interests

The authors declare that they have no competing interests.

## Authors' contributions

JQ and HX carried out all experimental procedures. BJN conceived and designed the study, wrote and guided the editing of the manuscript. All authors read and approved the final manuscript.

## References

[B1] Linos E Swetter SM Cockburn MG Colditz GA Clarke CA Increasing burden of melanoma in the United States J Invest Dermatol 2009 129 1666 1674 10.1038/jid.2008.423 19131946 PMC2866180

[B2] Jemal A Siegel R Ward E Hao Y Xu J Murray T Thun MJ Cancer statistics, 2008 CA Cancer J Clin 2008 58 71 96 10.3322/CA.2007.0010 18287387

[B3] Davies H Bignell GR Cox C Stephens P Edkins S Clegg S Teague J Woffendin H Garnett MJ Bottomley W Mutations of the BRAF gene in human cancer Nature 2002 417 949 954 10.1038/nature00766 12068308

[B4] Tuveson DA Weber BL Herlyn M BRAF as a potential therapeutic target in melanoma and other malignancies Cancer Cell 2003 4 95 98 10.1016/S1535-6108(03)00189-2 12957284

[B5] Wan PT Garnett MJ Roe SM Lee S Niculescu-Duvaz D Good VM Jones CM Marshall CJ Springer CJ Barford D Marais R Mechanism of activation of the RAF-ERK signaling pathway by oncogenic mutations of B-RAF Cell 2004 116 855 867 10.1016/S0092-8674(04)00215-6 15035987

[B6] Flaherty KT Yasothan U Kirkpatrick P Vemurafenib Nat Rev Drug Discov 2011 10 811 812 10.1038/nrd3579 22037033

[B7] Ji Z Flaherty KT Tsao H Targeting the RAS pathway in melanoma Trends Mol Med 2012 18 27 35 10.1016/j.molmed.2011.08.001 21962474 PMC3759017

[B8] Bollag G Hirth P Tsai J Zhang J Ibrahim PN Cho H Spevak W Zhang C Zhang Y Habets G Clinical efficacy of a RAF inhibitor needs broad target blockade in BRAF-mutant melanoma Nature 2010 467 596 599 10.1038/nature09454 20823850 PMC2948082

[B9] Flaherty KT Puzanov I Kim KB Ribas A McArthur GA Sosman JA O'Dwyer PJ Lee RJ Grippo JF Nolop K Chapman PB Inhibition of mutated, activated BRAF in metastatic melanoma N Engl J Med 2010 363 809 819 10.1056/NEJMoa1002011 20818844 PMC3724529

[B10] Hatzivassiliou G Song K Yen I Brandhuber BJ Anderson DJ Alvarado R Ludlam MJ Stokoe D Gloor SL Vigers G RAF inhibitors prime wild-type RAF to activate the MAPK pathway and enhance growth Nature 2010 464 431 435 10.1038/nature08833 20130576

[B11] Johannessen CM Boehm JS Kim SY Thomas SR Wardwell L Johnson LA Emery CM Stransky N Cogdill AP Barretina J COT drives resistance to RAF inhibition through MAP kinase pathway reactivation Nature 2010 468 968 972 10.1038/nature09627 21107320 PMC3058384

[B12] Joseph EW Pratilas CA Poulikakos PI Tadi M Wang W Taylor BS Halilovic E Persaud Y Xing F Viale A The RAF inhibitor PLX4032 inhibits ERK signaling and tumor cell proliferation in a V600E BRAF-selective manner Proc Natl Acad Sci USA 2010 107 14903 14908 10.1073/pnas.1008990107 20668238 PMC2930420

[B13] Nazarian R Shi H Wang Q Kong X Koya RC Lee H Chen Z Lee MK Attar N Sazegar H Melanomas acquire resistance to B-RAF(V600E) inhibition by RTK or N-RAS upregulation Nature 2010 468 973 977 10.1038/nature09626 21107323 PMC3143360

[B14] Poulikakos PI Zhang C Bollag G Shokat KM Rosen N RAF inhibitors transactivate RAF dimers and ERK signalling in cells with wild-type BRAF Nature 2010 464 427 430 10.1038/nature08902 20179705 PMC3178447

[B15] Pratilas CA Taylor BS Ye Q Viale A Sander C Solit DB Rosen N (V600E)BRAF is associated with disabled feedback inhibition of RAF-MEK signaling and elevated transcriptional output of the pathway Proc Natl Acad Sci USA 2009 106 4519 4524 10.1073/pnas.0900780106 19251651 PMC2649208

[B16] Smalley KS A pivotal role for ERK in the oncogenic behaviour of malignant melanoma? Int J Cancer 2003 104 527 532 10.1002/ijc.10978 12594806

[B17] Cooper JA Hunter T Major substrate for growth factor-activated protein-tyrosine kinases is a low-abundance protein Mol Cell Biol 1985 5 3304 3309 3879813 10.1128/mcb.5.11.3304-3309.1985 PMC369150

[B18] Kohno M Diverse mitogenic agents induce rapid phosphorylation of a common set of cellular proteins at tyrosine in quiescent mammalian cells J Biol Chem 1985 260 1771 1779 3871438

[B19] Rossomando AJ Payne DM Weber MJ Sturgill TW Evidence that pp 42, a major tyrosine kinase target protein, is a mitogen-activated serine/threonine protein kinase Proc Natl Acad Sci USA 1989 86 6940 6943 10.1073/pnas.86.18.6940 2550926 PMC297966

[B20] Boulton TG Nye SH Robbins DJ Ip NY Radziejewska E Morgenbesser SD DePinho RA Panayotatos N Cobb MH Yancopoulos GD ERKs: a family of protein-serine/threonine kinases that are activated and tyrosine phosphorylated in response to insulin and NGF Cell 1991 65 663 675 10.1016/0092-8674(91)90098-J 2032290

[B21] Pages G Guerin S Grall D Bonino F Smith A Anjuere F Auberger P Pouyssegur J Defective thymocyte maturation in p44 MAP kinase (Erk 1) knockout mice Science 1999 286 1374 1377 10.1126/science.286.5443.1374 10558995

[B22] Yao Y Li W Wu J Germann UA Su MS Kuida K Boucher DM Extracellular signal-regulated kinase 2 is necessary for mesoderm differentiation Proc Natl Acad Sci USA 2003 100 12759 12764 10.1073/pnas.2134254100 14566055 PMC240691

[B23] McCubrey JA Steelman LS Abrams SL Lee JT Chang F Bertrand FE Navolanic PM Terrian DM Franklin RA D'Assoro AB Roles of the RAF/MEK/ERK and PI3K/PTEN/AKT pathways in malignant transformation and drug resistance Adv Enzyme Regul 2006 46 249 279 10.1016/j.advenzreg.2006.01.004 16854453

[B24] Madhunapantula SV Robertson GP Is B-Raf a good therapeutic target for melanoma and other malignancies? Cancer Res 2008 68 5 8 10.1158/0008-5472.CAN-07-2038 18172288

[B25] Sturgill TW MAP kinase: it's been longer than fifteen minutes Biochem Biophys Res Commun 2008 371 1 4 10.1016/j.bbrc.2008.04.002 18406346

[B26] Zhuang L Lee CS Scolyer RA McCarthy SW Palmer AA Zhang XD Thompson JF Bron LP Hersey P Activation of the extracellular signal regulated kinase (ERK) pathway in human melanoma J Clin Pathol 2005 58 1163 1169 10.1136/jcp.2005.025957 16254105 PMC1770768

[B27] Lejeune FJ Rimoldi D Speiser D New approaches in metastatic melanoma: biological and molecular targeted therapies Expert Rev Anticancer Ther 2007 7 701 713 10.1586/14737140.7.5.701 17492933

[B28] Solit DB Rosen N Resistance to BRAF inhibition in melanomas N Engl J Med 2011 364 772 774 10.1056/NEJMcibr1013704 21345109

[B29] Jayaraman S Flow cytometric determination of mitochondrial membrane potential changes during apoptosis of T lymphocytic and pancreatic beta cell lines: comparison of tetramethylrhodamineethylester (TMRE), chloromethyl-X-rosamine (H2-CMX-Ros) and MitoTracker Red 580 (MTR580) J Immunol Methods 2005 306 68 79 10.1016/j.jim.2005.07.024 16256133

[B30] Qin JZ Xin H Sitailo LA Denning MF Nickoloff BJ Enhanced killing of melanoma cells by simultaneously targeting Mcl-1 and NOXA Cancer Res 2006 66 9636 9645 10.1158/0008-5472.CAN-06-0747 17018621

[B31] Qin JZ Ziffra J Stennett L Bodner B Bonish BK Chaturvedi V Bennett F Pollock PM Trent JM Hendrix MJ Proteasome inhibitors trigger NOXA-mediated apoptosis in melanoma and myeloma cells Cancer Res 2005 65 6282 6293 10.1158/0008-5472.CAN-05-0676 16024630

[B32] Dhomen N Marais R New insight into BRAF mutations in cancer Curr Opin Genet Dev 2007 17 31 39 10.1016/j.gde.2006.12.005 17208430

[B33] Sheridan C Brumatti G Elgendy M Brunet M Martin SJ An ERK-dependent pathway to Noxa expression regulates apoptosis by platinum-based chemotherapeutic drugs Oncogene 2010 29 6428 6441 10.1038/onc.2010.380 20802529

[B34] Paraiso KH Fedorenko IV Cantini LP Munko AC Hall M Sondak VK Messina JL Flaherty KT Smalley KS Recovery of phospho-ERK activity allows melanoma cells to escape from BRAF inhibitor therapy Br J Cancer 2010 102 1724 1730 10.1038/sj.bjc.6605714 20531415 PMC2883709

[B35] Lin J Goto Y Murata H Sakaizawa K Uchiyama A Saida T Takata M Polyclonality of BRAF mutations in primary melanoma and the selection of mutant alleles during progression Br J Cancer 2011 104 464 468 10.1038/sj.bjc.6606072 21224857 PMC3049568

[B36] Friday BB Yu C Dy GK Smith PD Wang L Thibodeau SN Adjei AA BRAF V600E disrupts AZD6244-induced abrogation of negative feedback pathways between extracellular signal-regulated kinase and Raf proteins Cancer Res 2008 68 6145 6153 10.1158/0008-5472.CAN-08-1430 18676837

[B37] Ferrell JE Jr Self-perpetuating states in signal transduction: positive feedback, double-negative feedback and bistability Curr Opin Cell Biol 2002 14 140 148 10.1016/S0955-0674(02)00314-9 11891111

[B38] Mansour SJ Resing KA Candi JM Hermann AS Gloor JW Herskind KR Wartmann M Davis RJ Ahn NG Mitogen-activated protein (MAP) kinase phosphorylation of MAP kinase kinase: determination of phosphorylation sites by mass spectrometry and site-directed mutagenesis J Biochem 1994 116 304 314 7822248 10.1093/oxfordjournals.jbchem.a124524

[B39] Bermudez O Pages G Gimond C The dual-specificity MAP kinase phosphatases: critical roles in development and cancer Am J Physiol Cell Physiol 299 C189 202 10.1152/ajpcell.00347.2009 20463170

[B40] Brummer T Naegele H Reth M Misawa Y Identification of novel ERK-mediated feedback phosphorylation sites at the C-terminus of B-Raf Oncogene 2003 22 8823 8834 10.1038/sj.onc.1207185 14654779

[B41] Dougherty MK Muller J Ritt DA Zhou M Zhou XZ Copeland TD Conrads TP Veenstra TD Lu KP Morrison DK Regulation of Raf-1 by direct feedback phosphorylation Mol Cell 2005 17 215 224 10.1016/j.molcel.2004.11.055 15664191

[B42] Balmanno K Cook SJ Tumour cell survival signalling by the ERK1/2 pathway Cell Death Differ 2009 16 368 377 10.1038/cdd.2008.148 18846109

[B43] Puzanov I Burnett P Flaherty KT Biological challenges of BRAF inhibitor therapy Mol Oncol 2011 5 116 123 10.1016/j.molonc.2011.01.005 21393075 PMC5528282

[B44] Nissan MH Solit DB The "SWOT" of BRAF inhibition in melanoma: RAF inhibitors, MEK inhibitors or both? Curr Oncol Rep 2011 13 479 487 10.1007/s11912-011-0198-4 21997758

[B45] Retsas S Latest developments in the treatment of melanoma: 'a penicillin moment for cancer'? J R Soc Med 2011 104 269 272 10.1258/jrsm.2011.100405 21659402 PMC3110965

